# A harsher reality for adolescents with depression on social media

**DOI:** 10.1038/s41598-025-89762-y

**Published:** 2025-03-31

**Authors:** Loes H. C. Janssen, Patti M. Valkenburg, Loes Keijsers, Ine Beyens

**Affiliations:** 1 Amsterdam School of Communication Research (ASCoR), University of Amsterdam, Amsterdam, the Netherlands; 2 Department of Psychology, Education, and Child Studies, Erasmus University Rotterdam, Rotterdam, the Netherlands

**Keywords:** Adolescents, Social media, Depressive symptoms, Depression, Social media experiences, Psychology, Human behaviour

## Abstract

Social media use is often highlighted as an important cause of the recent rise in depression among adolescents. However, this perspective overlooks a crucial reverse causality, namely that levels of depression might also shape adolescents’ social media use. In a diary study among 479 adolescents (*M*_*age*_ = 15.98; 16.9% clinically depressed), we assessed their level of depression and then monitored their social media activities and experiences daily for 100 subsequent days. Depressed adolescents did not differ from their non-depressed peers in the frequency of posting and the time spent scrolling. However, they reported feeling twice as insecure after scrolling, nearly twice as rejected during online communication with friends, and significantly more preoccupied with feedback. Our findings underscore the need for further research into the mechanisms of how depressive symptoms influence adolescents’ social media experiences, to inform the development of effective digital tools and therapeutic approaches to support adolescent mental health.

**Supplementary Information:**

The online version contains supplementary material available at 10.1038/s41598-025-89762-y.

## A harsher reality for adolescents with depression on social media

Over the past decade, adolescents worldwide have experienced a notable increase in the prevalence of depressive symptoms^1,2^, from 24% in 2001–2010 to 37% in 2011–2020^2^. These symptoms include feelings of sadness, diminished interest or pleasure in activities, irritability, feelings of worthlessness, and problems with eating, sleeping, and concentrating^3^. The recent rise in depressive symptoms has increasingly been attributed to the simultaneous increase in social media use^1^. More than 60 empirical studies, with the majority being cross-sectional studies, have examined the relationship of social media with depression or depressive symptoms in adolescents (see for meta-analyses and reviews^4–7^). Although umbrella reviews reveal that higher levels of social media use are associated with higher levels of depressive symptoms, they typically conclude that these associations are small^8,9^.

It may be intuitive to conclude that social media use causes depression. Still, two important gaps in our understanding of the link between social media use and depressive symptoms make such conclusion premature. First, empirical studies and the public debate have largely disregarded an alternative hypothesis for this link, namely that adolescents’ depressive symptoms may shape their social media activities, that is, posting, communicating with friends, and scrolling. The majority of research, both empirical studies and reviews, treat social media as the cause of depressive symptoms rather than a potential consequence. With some notable exceptions^10–12^, this social media effects view is even predominant in longitudinal studies that allow for the investigation of reciprocal effects (e.g^13–15^). This is surprising given that it is highly likely that depressive symptoms influence how adolescents engage in social media activities^16–18^. Hence, the main purpose of this study is to explore a quite plausible alternative hypothesis, that adolescents with more depressive symptoms engage in social media activities differently than adolescents with fewer symptoms.

A second major empirical gap in current research is that adolescents’ specific experiences on social media have been largely overlooked. Most studies have concentrated on the *quantity* of social media activities in relation to depressive symptoms, such as the time spent posting, communicating with friends, or scrolling. Such assessments fail to account for adolescents’ affective and cognitive appraisals of social media activities^19–22^. These appraisals are especially crucial in the context of depressive symptoms since these symptoms can profoundly (and negatively) impact how adolescents process social stimuli and interactions^23–26^ and interpret events^27^. Adolescents with more depressive symptoms may have different positive and negative social media experiences than adolescents with fewer depressive symptoms. For example, adolescents with more depressive symptoms may experience communication with friends online as less supportive and more rejecting than adolescents with fewer depressive symptoms. Therefore, the current study aims to provide unique insights into how adolescents with depressive symptoms may *experience* social media activities differently than adolescents without such symptoms.

This study focuses on the three primary social media activities identified in the literature: self-presentation through posting, online communication with friends, and scrolling content^20,28,29^. Adolescents with depressive symptoms may experience these activities differently. Firstly, posting allows adolescents to express or present themselves^29^ and seek peer approval^30^. After posting, they may be preoccupied with others’ responses, such as likes^31,32^, and adolescents with depressive symptoms might feel more preoccupied after posting with the responses than their peers with fewer depressive symptoms^33^. Their perception of feedback positivity may also vary based on their depressive symptoms. Secondly, during online communication with friends, adolescents may either receive support from friends^34,35^ or feel ignored or rejected^20^. Interpersonal theories of depression (e.g.,^26^), suggest that negative self-statements and excessive reassurance seeking of adolescents with depressive symptoms may lead to a reduction or withdrawal of support from others. Thirdly, when scrolling social media content, adolescents may seek to escape their depressive feelings by finding humorous content^20,34^ but may also compare themselves to others’ seemingly perfect lives, which may evoke feelings of insecurity^36^.

To capture adolescents’ actual lived experiences rather than their retrospective remembered experiences^37^, which could be impacted by their depressive symptoms, we employed a 100-day diary study among 479 adolescents. One week before the start of the diary study, we asked adolescents to report on their depressive symptoms (using the RADS-2^38^). For 100 subsequent days, adolescents indicated daily whether they had posted on social media, communicated online with their friends, and scrolled through social media. If they had, they answered questions about the quantity of these activities as well as their positive and negative experiences with these activities (i.e., feedback preoccupation, positivity of feedback, friend support, friend rejection, scrolling-induced fun, and scrolling-induced insecurity).

We investigated whether adolescents’ depressive symptoms correlated with subsequent **quantity** of social media **activities** in terms of posting frequency, time spent communicating online with friends, and time spent scrolling (RQ1a). We also examined whether adolescents’ depressive symptoms correlated with their social media **experiences** in terms of feedback preoccupation, positivity of feedback, friend support, friend rejection, scrolling-induced fun, and scrolling-induced insecurity (RQ1b). Finally, we examined how much within-group variation in social media activities and experiences exists among adolescents with clinically significant levels and non-clinically significant levels of depressive symptoms (using a clinical cut-off; RQ2). As the current study is, to our knowledge, the first to empirically examine these positive and negative social media experiences, we refrained from formulating hypotheses.

## Results

### Descriptives statistics and correlations

In this preregistered study (https://osf.io/nmj5b/) 479 adolescents participated (*M*_*age*_ = 15.98, *SD* = 1.15; 44.3% boys, 54.9% girls, 0.8% non-binary). Table 1 displays the descriptive statistics of study variables and between-person Pearson correlation coefficients with depressive symptoms (see Table S1 for full correlation table in the Supplementary Materials). All youth (479; 100%) used social media. Posting on social media, at least once across the study period, was done by most adolescents (410; 85.6%). Adolescents communicated online with friends for almost an hour a day (on average), with a maximum average of 4 h a day. Adolescents scrolled (watching photos, clips, or videos on social media) on average for two hours a day, with a maximum of eleven and a half hours a day.

**Table 1:** Descriptive statistics and between-person correlations of study variables.

Variables	Descriptives			Correlation with depressive symptoms
	*n*	*M*	SD	
Depressive symptoms	479	19.77	5.79	
Quantity social media activities (RQ1a)				
Frequency of social media posting	479	0.30	0.32	0.019
Time spent communicating online with friends	479	59.10	47.56	0.089
Time spent scrolling on social media	479	120.37	66.35	0.090*
Social media experiences (RQ1b)				
Feedback preoccupation	410	26.82	19.69	0.223***
Perceived positivity of feedback	410	66.67	16.19	− 0.171***
Friend support in online communication	479	70.21	13.94	− 0.209***
Friend rejection in online communication	479	18.03	14.79	0.405***
Scrolling-induced fun	479	68.32	16.33	− 0.134**
Scrolling-induced insecurity	479	20.02	20.13	0.446***

*p < .05; **p < .01; ***p < .001.

To answer RQ1a, we assessed whether adolescents’ depressive symptoms were related to the quantity of social media activities. Adolescents’ depressive symptoms were not related to the frequency of social media posting (*r* = .019, *p* = .681) or to the time spent communicating online with friends (*r* = .089, *p* = .051). However, adolescents with more depressive symptoms reported spending more time scrolling on social media (*r* = .090, *p* = .049) than adolescents with fewer depressive symptoms.

In terms of social media experiences (RQ1b), adolescents with more depressive symptoms perceived the feedback on their posts as less positive (*r* = − .171, *p* < .001), felt less supported by their friends in online communication (*r* = − .209, *p* < .001), and had less fun while scrolling (*r* = − .134, *p* = .003) compared to adolescents with fewer depressive symptoms. Adolescents with more depressive symptoms were also more preoccupied with feedback on their posts (*r* = .223, *p* < .001), felt more rejected by their friends in online communication (*r* = .405, *p* < .001), and experienced more insecurity while scrolling (*r* = .446, *p* < .001).

## Differences between depressed versus non-depressed adolescents

Using the established cut-off score of 26 on the depressive symptom scale^39^, we categorized adolescents into two groups to also assess whether depressed versus non-depressed adolescents would differ. Results revealed that 16.9% of adolescents (*n* = 81) reported clinically significant levels of depressive symptoms (labeled “depressed adolescents”), while 83.1% (*n* = 398) did not report such levels of depressive symptoms (labeled “non-depressed adolescents”). Descriptive statistics and between-person correlations for depressed adolescents are presented in Table S2 and for non-depressed adolescents in Table S3 in the Supplementary Materials.

**Fig. 1:**
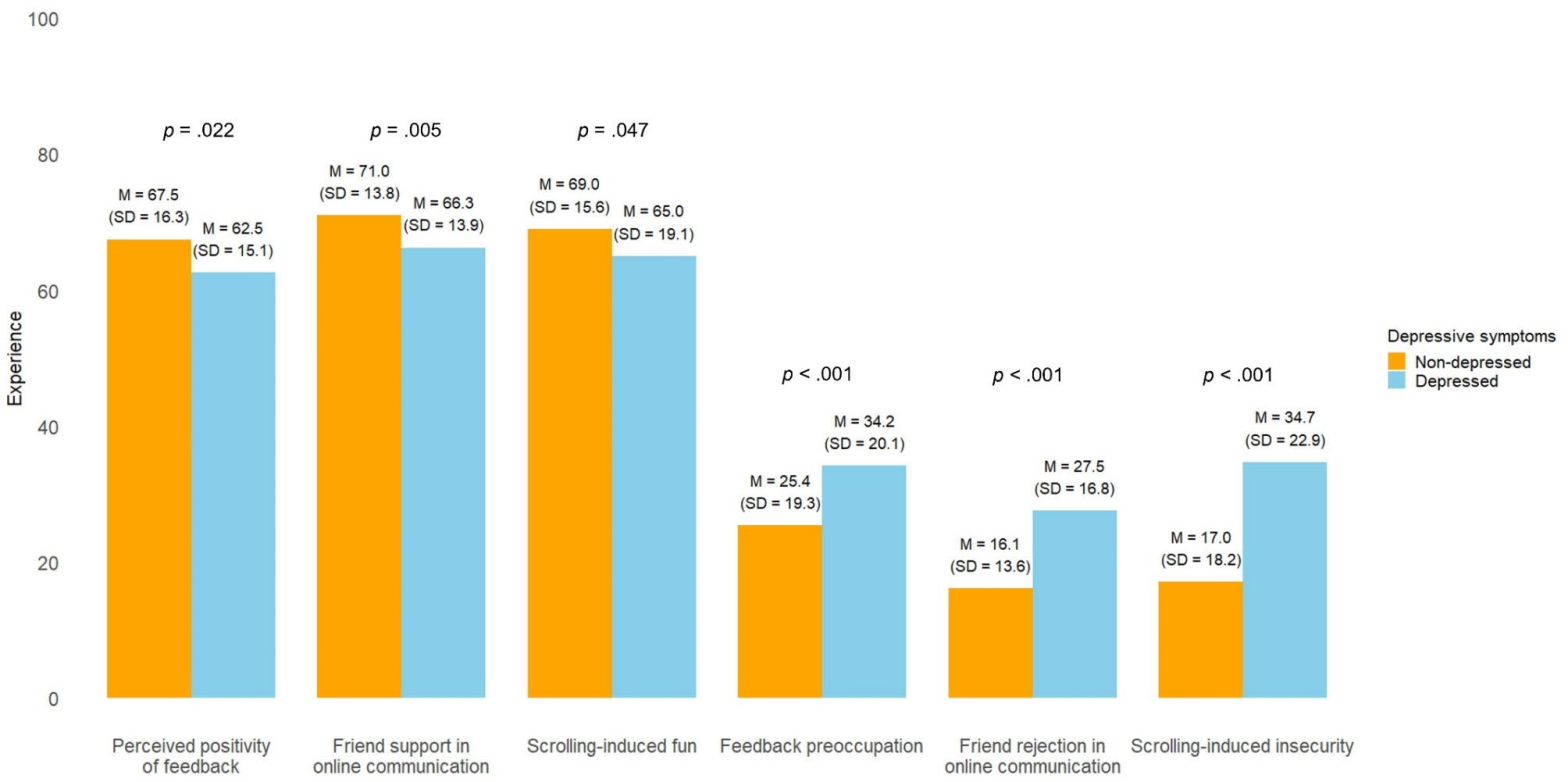
Differences between depressed (*n* = 81) vs. non-depressed adolescents (*n* = 398) in positive and negative social media experiences. Means per group are displayed. Means differed significantly for all variables using ANOVA tests.

We first compared the groups to each other (Fig. 1). Depressed adolescents reported less positive and more negative social media experiences than non-depressed adolescents. The differences between the depressed and non-depressed adolescents were most pronounced in their negative social media experiences, feedback preoccupation, friend rejection in online communication, and scrolling-induced insecurity. Depressed adolescents reported twice as much scrolling-induced insecurity (*M* = 34.7, *SD* = 22.9) compared to their non-depressed adolescents (*M* = 17.0, *SD* = 18.2). They also felt nearly twice as often rejected during online communication with friends (*M* = 27.5, *SD* = 16.8) compared to non-depressed adolescents (*M* = 16.1, *SD* = 13.6). Finally, feedback preoccupation was approximately one-third higher among depressed adolescents (*M* = 34.2, *SD* = 20.1) than non-depressed adolescents (*M* = 25.4, *SD* = 19.3).

To explore whether depressed adolescents differed more from each other than from the non-depressed adolescents in social media activities and experiences (RQ2), we compared the mean sum of squares between and within groups (see Table 2). Concerning the quantity of social media activities, the variation in time spent communicating with friends online was larger between the two groups (MSB = 0.924) than within the groups (MSW = 0.139). However, for the frequency of social media posting and time spent scrolling on social media, the variation was larger within than between the groups. For all social media experiences, the variation between the two groups was larger than within the groups. Together, these findings indicate that depressed adolescents differed more from non-depressed adolescents in time spent communicating online with friends and their social media experiences than from each other. With regard to the frequency of social media posting and time spent scrolling on social media, depressed adolescents differed more from other depressed adolescents than from non-depressed adolescents. The preregistered sum of squares, which were deemed less appropriate as these are influenced by the different sample sizes of the two groups, are.

reported in Table S4 in the Supplementary Materials. Violin plots showing the distributions of the quantity of social media activities and the positive experiences among depressed and non-depressed adolescents are presented in Fig. S1 to S4 in the Supplementary Materials.

**Table 2:** Differences between depressed and non-depressed adolescents regarding the quantity of Social Media activities and positive and negative experiences.

Variable	MSB	MSW
Quantity social media activities		
Frequency of social media posting	0.000	0.141
Time spent communicating online with friends	0.924	0.139
Time spent scrolling on social media	0.042	0.141
Social media experiences		
Feedback preoccupation	1.522	0.134
Perceived positivity of feedback	0.714	0.136
Friend support in online communication	1.104	0.139
Friend rejection in online communication	5.649	0.129
Scrolling-induced fun	0.554	0.140
Scrolling-induced insecurity	7.282	0.126

MSB = Mean sum of squares between groups, MSW = Mean sum of squares within groups.

## Sensitivity analyses

To test the rigor of the findings sensitivity analyses excluding 1,004 potentially untrustworthy observations of the 44,211 completed observations (2.3%) were performed (see preregistration for details: https://osf.io/nmj5b/). These confirmed all conclusions with regard to the social media experiences. However, adolescents’ depressive symptoms were no longer related to time spent scrolling on social media (see Table S5 for descriptive statistics and between-person correlations with depressive symptoms in the Supplementary Materials).

In addition to our preregistered sensitivity analyses, we tested whether gender and age could be confounders. Partial correlations demonstrated very similar results compared to the main analyses when controlling for gender and age. When controlling for age, the association between depressive symptoms and time spent scrolling on social media was no longer significant (*r* = .090, *p* = .050; see Table S6 in the Supplementary Materials for correlations). When controlling for age, one additional association was found: Adolescents with more depressive symptoms spent slightly more time communicating online with friends (*r* = .105, *p* = .021; see Table S7 in the Supplementary Materials for correlations).

## Discussion

In the vivid debate regarding the causal influence of social media on youths’ mental health, one crucial alternative hypothesis is typically ignored: Adolescents’ depressive symptoms may also negatively shape their social media experiences^16–18,23–27^. Our findings provide compelling evidence for this alternative perspective, revealing that depressed adolescents experience the online reality as harsher compared to non-depressed adolescents, with depressed adolescents having more negative and less positive social media experiences than their non-depressed peers. Specifically, depressed adolescents experienced twice as much insecurity after scrolling and nearly twice as much rejection from friends while communicating online. These unprecedently strong effects underscore the importance of considering how existing depressive symptoms influence adolescents’ experiences on social media.

Our 100-day diary approach strengthens our results and conclusions. By assessing adolescents’ depressive symptoms before tracking their social media activities and experiences over a 100-day diary period, we mitigated a potential negativity bias that depressive symptoms can have on how adolescents recall and interpret events^27^. Our approach allowed us to capture adolescents’ real-time experiences rather than their retrospective recollections. Consequently, our study shows the need to move beyond the traditional social media effects view and retrospective designs to gain a more nuanced understanding of the relation between adolescents’ depressive symptoms and social media.

Previous research has predominantly focused on the quantity of social media activities, largely neglecting adolescents’ appraisal of these activities^19–22^. Our results reveal that adolescents with more depressive symptoms consistently reported having more negative social media experiences than their peers with fewer depressive symptoms. Adolescents’ levels of depressive symptoms were inconsistently and weakly related to the frequency of posting, the time spent communicating with friends online, and the time spent scrolling. This suggests that while adolescents with more depressive symptoms spend an equal amount of time on social media activities as those with fewer depressive symptoms, their experiences are markedly different, leading to different online lives. Therefore, it is essential to take adolescents’ social media experiences into account.

Our findings align with existing studies that highlight the increased vulnerability of adolescents with mental health problems to have adverse experiences on social media^40,41^. For instance, a study found that adolescents who experienced self-harm, eating disorders, and attention deficit hyperactivity disorder (ADHD) were more likely to have negative online experiences, such as cyberbullying, than their peers^40^. Another important identified risk factor for adolescents with mental health problems is scrolling through overly positive content, which can trigger social comparison and evoke feelings of insecurity^41^. This result was supported in our study, as we found that depressed adolescents experienced twice as much scrolling-induced insecurity. While earlier research suggested that social media could provide greater opportunities for adolescents with more depressive symptoms or depression to seek and receive support^41^, our results suggest that these adolescents experienced less support than adolescents with less depressive symptoms or levels of depression as all their social media experiences were more negative.

The demonstrated relevance of these experiences for adolescents has important practical implications. It may be that adolescents’ negative appraisals of social media activities can create a harmful self-reinforcing feedback loop. For example, a depressed adolescent who seeks support from friends online may feel unsupported and rejected which may exacerbate the depressive symptoms^34^. To help adolescents break this cycle, it is crucial to raise awareness among those with depressive symptoms about their heightened risk of having negative social media experiences. Parents and clinicians could for instance discuss this self-reinforcing loop with adolescents, helping them cope with these negative experiences, and become more aware of the potential negativity bias. Clinicians could also use psychoeducation combined with social media literacy training to explain the nature of the negativity bias and how social media may worsen this^42^.

Parents and clinicians may also advise a more selective social media diet, taking into consideration the positive experiences that adolescents may also have, offering a tailored approach. While our findings indicated that the differences between depressed and non-depressed adolescents are larger than the differences within each group, differences across depressed adolescents still exist, which aligns with qualitative work in depressed adolescents^34^. Some depressed adolescents may experience more friend rejection and less support from friends, while others experience more friend rejection but also *more* support from friends. Parents or clinicians could talk with adolescents and distinguish which positive social media experiences they have, how they may be amplified, and how negative social media experiences may be reduced. Clinicians may use a diary approach and ask adolescents to keep track of their experiences, mitigating a recall bias, to unravel which social media experiences may be positive for adolescents.

Similar suggestions could be applied in a school setting. Teachers could provide adolescents with social media literacy education, including lessons on how adolescents’ mood or psychological symptoms may influence their social media use and experiences. Such lessons could equip adolescents with tools to promote more mindful online behavior. Earlier work indicated the potential effectiveness of these types of interventions related to body image, media literacy, and understanding of advertising^43,44^. Additionally, teachers may use classroom activities or projects for which adolescents must track their social media use. This could enhance self-awareness and help them reflect on positive and negative social media experiences. Sharing these reflections in a classroom setting could enable adolescents to benefit from each other’s insights and strategies.

This study is one of the first to assess adolescents’ experiences across the three primary social media activities (i.e., posting, communicating with friends, and scrolling) while using a 100-day diary design to minimize recall bias. However, it is important to acknowledge that we still relied on adolescents’ self-reports. Adolescents may differ in their capacity to reflect on their emotions and behaviors (i.e., introspective ability), which may have influenced our findings. To reduce such potential bias, we piloted the questions assessing the social media experiences to ensure they were concrete and specific, and we limited the recall period to one day to improve precision. Despite these precautions, capturing subjective experiences like emotions and appraisals inherently depends on self-reports, making it difficult to fully eliminate an introspective bias.

Another limitation of our study is the lack of insights into the specific content that adolescents encountered on social media, which may have been more negative for adolescents with depression. Some adolescents with depression may actively search for information about depression or videos of shared experiences but inadvertently come across related harmful or triggering content^34^. Given that social media platforms use algorithms to tailor the content to individuals’ preferences^45^, this may increase the risk of exposure to more harmful or negative content for these adolescents^40^. Future studies could benefit from using for instance data donations, where adolescents provide access to their social media usage data. This approach would offer valuable insight into the specific content adolescents encounter and enable researchers to link this to their social media experiences.

Additionally, future research may want to explore whether depressive symptoms impact adolescents’ social experiences similarly across both online and offline contexts. Our finding that adolescents with depression experienced more friend rejection in online communication aligns with earlier work showing that adolescents with higher levels of depressive symptoms reported more peer rejection offline than adolescents with lower levels of depressive symptoms^46^. Examining both contexts simultaneously is crucial for understanding how experiences in one context may influence and impact the other. For instance, some adolescents prefer seeking emotional support in person rather than online^47^, while others may favor online interactions. Particularly, those with more depressive symptoms tend to favor communicating with people online rather than in person compared to those with little to no symptoms^48^.

A final suggestion for future research is to investigate how positive social (media) experiences may mitigate negative experiences. Qualitative studies have shown that adolescents describe both positive and negative experiences when using social media^20,22^, including those with depression^34^. However, it remains unclear whether positive experiences, such as receiving support in online communication with friends, may counteract negative experiences like rejection in the same context. Future studies should focus on exploring these dynamics to provide more detailed insights. This approach could ultimately lead to more tailored clinical interventions and advice aimed at improving adolescents’ mental health and experiences.

To conclude, our study stresses the urgent need to expand the societal debate from viewing social media solely as a cause of depressive symptoms to a more nuanced perspective that recognizes that adolescents with depressive symptoms *experience* social media activities much more negatively. Specifically, higher levels of depressive symptoms precede more negative and fewer positive social media experiences, and we suggest that this may set into motion a negative feedback loop of increasing depressive feelings. Embracing this alternative hypothesis and considering both positive and negative social media experiences would enable gaining more nuanced insights into the relation between depressive symptoms and social media. It would also facilitate discussions on strategies to effectively support at-risk adolescents, ultimately fostering better mental health outcomes.

## Methods

This preregistered study (https://osf.io/nmj5b/) is part of a larger intensive longitudinal project that investigates the effects of social media use on indicators of adolescent well-being. The data collection took place from January 2023 to June 2023 and consisted of four parts: (1) an online intake interview, (2) a baseline questionnaire, (3) a 100-day daily diary, and (4) an optional exit interview to provide insights into personal social media patterns for adolescents who were interested. The current study used data of the baseline questionnaire and 100-day daily diary.

The full project was approved by the Ethics Review Board of the Faculty of Social and Behavioral Sciences at the University of Amsterdam (2022-YME-15724) and was performed in accordance with the guidelines formulated by the Ethics Review Board and the principles of the Declaration of Helsinki. Adolescents provided informed consent to participate and if adolescents were below the age of 16, parents also provided informed consent.

### Participants

Participants were recruited in collaboration with an insight and strategy bureau (CHOICE) that had access to several panels of adults and adolescents (above the age of 16) who regularly participate in research. Adolescents who indicated their interest in participating were asked to invite friends. Moreover, participants were recruited from earlier projects, through social media, and the personal network of the researchers. A total of 480 adolescents aged 14 to 18 at the time of inclusion from all regions of the Netherlands started the larger project but one adolescent dropped out after the first day of the daily diary. The final sample consisted of 479 adolescents who participated in the 100-day diary study (*M*_*age*_ = 15.98, *SD* = 1.15; 44.3% boys, 54.9% girls, 0.8% non-binary). Adolescents were enrolled in (pre)vocational education (29.9%), higher general secondary/higher professional education (29.2%), and (preparatory) academic education (40.9%). The majority of adolescents were born in the Netherlands (96.9%).

### Procedure

A detailed overview of the project procedure, including the design of the study can be found on OSF (https://osf.io/k47ta/). The study procedure of the project was designed following recommendations for collecting intensive longitudinal data^49^. After providing consent, participants were invited for an online intake interview and received instructions for installing the daily diary software application m-Path (m-path.io^50^) on their smartphones. During the intake interviews, participants were asked about their own social media use, received more information about the study procedure, and practiced with the daily diary app. Five days before the 100-day diary study started, participants received a link to the baseline questionnaire, including questions about demographics and depressive symptoms.

During the diary part of the project, from January to May 2023, participants received one questionnaire a day via the m-Path app for 100 consecutive days. The questionnaires were sent at 8.30 PM and could be started until midnight. If participants had not yet completed the questionnaire, reminders were sent at 9.15 PM and 10.00 PM. Each questionnaire consisted of 34–38 questions, depending on follow-up questions. At the end of the 100-day diary, participants could extend their participation for 15 days to catch up for missed days. Participants’ compliance was monitored daily and participants’ questions and problems were answered via WhatsApp, telephone, and e-mail. We also regularly messaged participants via WhatsApp to motivate them or check in if there were problems. For instance, we updated them on their weekly response rates and contacted them when they missed three subsequent questionnaires.

To minimize potential harms or risks for participants, we included a statement at the end of the baseline questionnaire that if adolescents experienced problems or mental health issues they could contact the Dutch mental health organization @ease. This organization offers anonymous support to adolescents. We could also consult an employee of @ease if adolescents mentioned problems to us. During the diary part, we were available for contact until 10 PM and adolescents were instructed that they could contact us when there were issues.

#### Incentives

For every part of the study, except for the optional interview, participants received compensation. Adolescents received €5,- for the intake interview, €5,- for completing the baseline questionnaire, and €1,- for every completed daily diary questionnaire. Participants who completed 100 questionnaires or more (including catch-up days) received an additional €10,-. Participants who completed 14 consecutive questionnaires in a row in the middle of the study (day 47 until 60) could earn a €5,- bonus. In addition, once a week (Tuesday) we raffled two times €25,- based on compliance of the previous week. Participants received their compensation every month of the study.

#### Compliance

In research using experience sampling methods (ESM; assessing moods and experiences multiple times a day for weeks) and daily diary designs, a key quality marker is compliance^49^. After the 100 days, 82.8% of the sent daily diary questionnaires were completed (39,598 of 47,847 observations). After the 15 catch-up days, adolescents completed a total of 44,211 questionnaires, indicating an average of 92.3 daily questionnaires (*SD* = 24.55, range 12–115). A small proportion of the daily diaries (115 questionnaires, < 0.3%) had irregularities or were not sent due to unforeseen technical issues with the m-Path application. Not answering questionnaires was due to technological factors (e.g., uploading errors) and human factors (e.g., being ill).

### Measures

#### Baseline

*Depressive symptoms*. A validated and often used short form of the Reynolds Adolescent Depression Scale Second Edition (RADS-2^38^) was used to assess adolescents’ depressive symptoms at the start of the study. The scale consists of 10 items (e.g., “In the past two weeks I was sad”) which were answered on a 4-point scale ranging from 1 (*never*) to 4 (*often*). One item, which was positively formulated, was reverse coded before calculating a sum score. Higher scores reflected higher levels of depressive symptoms and Cronbach’s alpha was 0.86. Sum scores could range between 10 and 40. A cut-off point of 26 has been suggested as classification for clinically significant levels of depressive symptoms^39^. Using this cut-off point, we calculated a new variable representing whether adolescents’ depressive symptoms scores indicated clinically significant levels of depressive symptoms (i.e., equal to or above the cut-off; labeled depressed) or non-clinically significant levels of depressive symptoms (i.e., below the cut-off; labeled non-depressed).

#### Daily diary

To assess the social media experiences with low recall bias, adolescents indicated for 100 days in a row, whether they had posted on social media (and how much), communicated online with their friends, and scrolled. If so, follow-up questions were asked. For each variable, we computed a person-specific mean representing the quantity of social media use or social media experience throughout the 3 month study period.

*Frequency of social media posting*. Each day, we assessed whether adolescents posted something on social media by asking them: “Did you share or post a (private) story today (e.g., on Instagram, Snapchat, BeReal)?” Adolescents could select one answer of four: (0) no, nothing, (1) 1 time, (2) 2 times, and (3) more than 2 times. A dummy variable was created to represent if adolescents posted that day (1) or not (0).

*Feedback preoccupation*. If adolescents had posted on social media, we assessed their level of feedback preoccupation by asking them: “Were you preoccupied today with how others would react to your (private) story or post?” If adolescents shared two or more posts, the item was shown in plural. Adolescents answered on a Visual Analog Scale (VAS) that ranged from 0 (*not preoccupied at all*) to 100 (*very preoccupied*).

*Perceived positivity of feedback*. If adolescents had posted on social media, we assessed their perceived positivity of peer feedback by asking them: “Were you satisfied with the responses you received on your (private) story or post today?” If adolescents shared two or more posts, the item was shown in plural. Adolescents answered on a Visual Analog Scale (VAS) that ranged from 0 (*not satisfied at all*) to 100 (*very satisfied*).

*Time spent communicating online with friends*. Each day, we assessed how much time adolescents had spent communicating online with friends by asking them: “How long did you chat with your friends today (snap, WhatsApp, DM)?”. Adolescents were instructed to estimate hours and minutes, which we recoded to minutes.

*Friend support in online communication*. If adolescents had communicated with their friends online, the experience of support by their friends was assessed by asking adolescents: “Did you feel supported by your friends while chatting today?” Adolescents answered on a Visual Analog Scale (VAS) that ranged from 0 (*not supported at all*) to 100 (*very supported*).

*Friend rejection in online communication*. If adolescents had communicated with their friends online, the experience of rejection by friends was assessed by asking adolescents: “Did you feel ignored by your friends today after sending a snap, app, or DM?” Adolescents answered on a Visual Analog Scale (VAS) that ranged from 0 (*not ignored at all*) to 100 (*very ignored*).

*Time spent scrolling on social media*. We assessed how much time adolescents had spent scrolling on social media by asking them: “How long did you watch photos, clips, or videos on social media today?”. Adolescents were instructed to estimate hours and minutes, which we recoded to minutes.

*Scrolling-induced fun*. If adolescents had scrolled on social media, the experience of funny content during scrolling was assessed by asking adolescents: “Did you find the photos, clips, or videos funny?” Adolescents answered on a Visual Analog Scale (VAS) that ranged from 0 (*not funny at all*) to 100 (*very funny*).

*Scrolling-induced insecurity*. If adolescents had scrolled on social media, the experience of insecurity during scrolling was assessed by asking adolescents: “Did you feel insecure about yourself today when you saw photos, clips or videos on social media?” Adolescents answered on a Visual Analog Scale (VAS) that ranged from 0 (*not insecure at all*) to 100 (*very insecure*).

### Preregistered statistical analysis plan

All of our preregistered analyses (https://osf.io/nmj5b/*)* were performed in R (Version 4.2.2). To answer our first research question and investigate whether adolescents’ depressive symptoms were correlated with subsequent quantity of social media activities (i.e., frequency of posting, time spent communicating online with friends, and time spent scrolling on social media; RQ1a) and adolescents’ social media experiences throughout the 100 days (i.e., social media-induced feedback preoccupation, positivity of feedback, friend support, friend rejection, scrolling-induced fun, and scrolling-induced insecurity; RQ1b), we calculated between-person Pearson correlations coefficients. We aimed to answer the second research question by examining how much within-group variation in social media quantity and experiences exists among depressed adolescents and non-depressed adolescents (using sum of squares between and within the two groups). The between sum of squares indicates how much of the total variation, referring to the overall variability in the data, is due to differences between group means, while the within-group sum of squares indicates how much of the total variation is due to variation within the groups. However, due to the different sample sizes of the two groups that influence the sum of squares, we additionally calculated the mean sum of squares between and within groups to account for these different sample sizes. This enables comparing within- and between-group variation. Additionally, we plotted violin plots to depict the distributions of data.

## Electronic supplementary material

Below is the link to the electronic supplementary material.

Supplementary Material 1

## Data Availability

A detailed overview of the project procedure, including the design of the study can be found on the Open Science Framework (OSF) (https://osf.io/k47ta/). More information on the measures and preregistered analyses can be found in the preregistration on OSF (https://osf.io/nmj5b/). The R syntax necessary to reproduce the analyses are publicly accessible at OSF: https://osf.io/kvy9j/. The code was checked and run by an independent co-pilot. The data underlying this article can be found on Figshare: 10.21942/uva.28458422.v1.
