# Supplementary material for: A harsher reality for adolescents with depression on social media
Source: Sci Rep. 2025 Mar 31;15:10947. doi: 10.1038/s41598-025-89762-y (PMC11955549; doi:10.1038/s41598-025-89762-y)
Supplement: Supplementary file 1 — Supplementary Material 1 [file 41598_2025_89762_MOESM1_ESM.docx]

Supplementary Materials for

**A Harsher Reality for Adolescents with Depression on Social Media**

Loes H. C. Janssen^1*^, Patti M. Valkenburg^1^, Loes Keijsers^2^, and Ine Beyens^1^

^1^ Amsterdam School of Communication Research (ASCoR), University of Amsterdam; Amsterdam, the Netherlands.

^2^ Department of Psychology, Education, and Child Studies, Erasmus University Rotterdam; Rotterdam, the Netherlands.

* Corresponding author. Email: l.h.c.janssen@uva.nl

**The file includes:**

Tables S1 to S7

Figures S1 to S4

| Variables | Descriptives | |  | Between-person correlations | | | | | | | | |
| --- | --- | --- | --- | --- | --- | --- | --- | --- | --- | --- | --- | --- |
|  |  |  |  |  |  |  |  |  |  |  |  |  |
|  | *n* | *M* (SD) |  | 1 | 2 | 3 | 4 | 5 | 6 | 7 | 8 | 9 |
| 1. Depressive symptoms | 479 | 19.77 (5.79) |  |  |  |  |  |  |  |  |  |  |
| **Quantity social media activities** |  |  |  |  |  |  |  |  |  |  |  |  |
| 2. Frequency of social media posting | 479 | 0.30 (0.32) |  | .019 |  |  |  |  |  |  |  |  |
| 3. Time spent communicating online with friends | 479 | 59.10 (47.56) |  | .089 | .104* |  |  |  |  |  |  |  |
| 4. Time spent scrolling on social media | 479 | 120.37 (66.35) |  | .090* | .016 | .081 |  |  |  |  |  |  |
| **Social media experiences** |  |  |  |  |  |  |  |  |  |  |  |  |
| 5. Feedback preoccupation | 410 | 26.82 (19.69) |  | .223*** | .036 | .017 | -.036 |  |  |  |  |  |
| 6. Perceived positivity of feedback | 410 | 66.67 (16.19) |  | -.171*** | .043 | .055 | -.042 | -.262*** |  |  |  |  |
| 7. Friend support in online communication | 479 | 70.21 (13.94) |  | -.209*** | .122** | .155*** | -.071 | -.116* | .448*** |  |  |  |
| 8. Friend rejection in online communication | 479 | 18.03 (14.79) |  | .405*** | .130** | .125** | .029 | .621*** | -.365*** | -.231*** |  |  |
| 9. Scrolling-induced fun | 479 | 68.32 (16.33) |  | -.134** | .084 | .089 | .071 | .016 | .251*** | .431*** | -.035 |  |
| 10. Scrolling-induced insecurity | 479 | 20.02 (20.13) |  | .446*** | .129** | .096* | .023 | .532*** | -.281*** | -.222*** | .733*** | -.089 |

**p* < .05; ***p* < .01; ****p* < .001

Table S1. Descriptive statistics and between-person correlations of depressive symptoms, quantity of social media activities, and social media experiences.

| Variables | Descriptives | |  | Between-person correlations | | | | | | | | |
| --- | --- | --- | --- | --- | --- | --- | --- | --- | --- | --- | --- | --- |
|  |  |  |  |  |  |  |  |  |  |  |  |  |
|  | *n* | *M* (SD) |  | 1 | 2 | 3 | 4 | 5 | 6 | 7 | 8 | 9 |
| 1. Depressive symptoms | 81 | 29.79 (3.07) |  |  |  |  |  |  |  |  |  |  |
| **Quantity social media activities** |  |  |  |  |  |  |  |  |  |  |  |  |
| 2. Frequency of social media posting | 81 | 0.30 (0.29) |  | -.009 |  |  |  |  |  |  |  |  |
| 3. Time spent communicating online with friends | 81 | 71.44 (53.48) |  | -.091 | -.071 |  |  |  |  |  |  |  |
| 4. Time spent scrolling on social media | 81 | 124.04 (62.34) |  | .116 | -.042 | .161 |  |  |  |  |  |  |
| **Social media experiences** |  |  |  |  |  |  |  |  |  |  |  |  |
| 5. Feedback preoccupation | 67 | 34.15 (20.07) |  | .002 | .044 | -.080 | -.197 |  |  |  |  |  |
| 6. Perceived positivity of feedback | 67 | 62.54 (15.11) |  | -.387** | .219 | .126 | -.013 | -.437*** |  |  |  |  |
| 7. Friend support in online communication | 81 | 66.26 (13.87) |  | -.273* | .212 | .188 | -.014 | -.418*** | .659*** |  |  |  |
| 8. Friend rejection in online communication | 81 | 27.52 (16.76) |  | .059 | .095 | -.038 | -.040 | .686*** | -.480*** | -.337** |  |  |
| 9. Scrolling-induced fun | 81 | 65.04 (19.14) |  | -.075 | .053 | .125 | .034 | -.091 | .263* | .499*** | .053 |  |
| 10. Scrolling-induced insecurity | 81 | 34.68 (22.88) |  | .123 | .093 | -.252* | .019 | .577*** | -.373** | -.475*** | .579*** | -.054 |

**p* < .05; ***p* < .01; ****p* < .001

Table S2. Descriptive statistics and between-person correlations of depressive symptoms, quantity of social media activities, and social media experiences for depressed adolescents

| Variables | Descriptives | |  | Between-person correlations | | | | | | | | |
| --- | --- | --- | --- | --- | --- | --- | --- | --- | --- | --- | --- | --- |
|  |  |  |  |  |  |  |  |  |  |  |  |  |
|  | *n* | *M* (SD) |  | 1 | 2 | 3 | 4 | 5 | 6 | 7 | 8 | 9 |
| 1. Depressive symptoms | 398 | 17.73 (3.71) |  |  |  |  |  |  |  |  |  |  |
| **Quantity social media activities** |  |  |  |  |  |  |  |  |  |  |  |  |
| 2. Frequency of social media posting | 398 | 0.30 (0.32) |  | .036 |  |  |  |  |  |  |  |  |
| 3. Time spent communicating online with friends | 398 | 56.59 (45.93) |  | .013 | .143** |  |  |  |  |  |  |  |
| 4. Time spent scrolling on social media | 398 | 119.62 (67.19) |  | .113* | .026 | .061 |  |  |  |  |  |  |
| **Social media experiences** |  |  |  |  |  |  |  |  |  |  |  |  |
| 5. Feedback preoccupation | 343 | 25.38 (19.33) |  | .179*** | .032 | .016 | -.015 |  |  |  |  |  |
| 6. Perceived positivity of feedback | 343 | 67.47 (16.29) |  | -.095 | .018 | .056 | -.042 | -.214*** |  |  |  |  |
| 7. Friend support in online communication | 398 | 71.02 (13.84) |  | -.160** | .107* | .170*** | -.079 | -.041 | -.405*** |  |  |  |
| 8. Friend rejection in online communication | 398 | 16.10 (13.59) |  | .353*** | .146** | .134** | .037 | .590*** | -.323*** | -.172*** |  |  |
| 9. Scrolling-induced fun | 398 | 68.99 (15.64) |  | -.107* | .093 | .093 | .083 | .063 | .242*** | .408*** | -.028 |  |
| 10. Scrolling-induced insecurity | 398 | 17.03 (18.15) |  | .367*** | .149** | .154** | .016 | .498*** | -.238*** | -.122* | .746*** | -.066 |

**p* < .05; ***p* < .01; ****p* < .001

Table S3. Descriptive statistics and between-person correlations of depressive symptoms, quantity of social media activities, and social media experiences for non-depressed adolescents

| Variable | SSB (df) | SSW (df) | *F* | *p* |
| --- | --- | --- | --- | --- |
| **Quantity social media activities** |  |  |  |  |
| Frequency of social media posting | 0.00 (1) | 67.30 (477) | 0.00 | .992 |
| Time spent communicating online with friends | 0.92 (1) | 66.38 (477) | 6.64 | .010 |
| Time spent scrolling on social media | 0.04 (1) | 67.26 (477) | 0.30 | .586 |
| **Social media experiences** |  |  |  |  |
| Feedback preoccupation | 1.52 (1) | 54.53 (408) | 11.39 | <.001 |
| Perceived positivity of feedback | 0.71 (1) | 55.34 (408) | 5.26 | .022 |
| Friend support in online communication | 1.10 (1) | 66.20 (477) | 7.95 | .005 |
| Friend rejection in online communication | 5.65 (1) | 61.65 (477) | 43.70 | <.001 |
| Scrolling-induced fun | 0.55 (1) | 66.75 (477) | 3.96 | .047 |
| Scrolling-induced insecurity | 7.28 (1) | 60.02 (477) | 57.87 | <.001 |

*Note*. SSB = Sum of squares between groups, SSW = Sum of squares within groups

Table S4. Sum of squares between and within groups of study variables with regard to the quantity of social media activities and positive and negative experiences

| Variables | Descriptives | | |  | Correlation with depressive symptoms |
| --- | --- | --- | --- | --- | --- |
|  | *n* | *M* | SD |  |  |
| Depressive symptoms | 479 | 19.77 | 5.79 |  |  |
| **Quantity social media activities** |  |  |  |  |  |
| Frequency of social media posting | 479 | 0.30 | 0.32 |  | .020 |
| Time spent communicating online with friends | 479 | 59.07 | 47.61 |  | .082 |
| Time spent scrolling on social media | 477 | 121.66 | 65.67 |  | .072 |
| **Social media experiences** |  |  |  |  |  |
| Feedback preoccupation | 409 | 26.64 | 19.65 |  | .232*** |
| Perceived positivity of feedback | 409 | 66.91 | 16.26 |  | -.182*** |
| Friend support in online communication | 479 | 70.33 | 13.94 |  | -.213*** |
| Friend rejection in online communication | 479 | 17.81 | 14.63 |  | .415*** |
| Scrolling-induced fun | 477 | 68.55 | 16.38 |  | -.135** |
| Scrolling-induced insecurity | 477 | 19.76 | 20.14 |  | .461*** |

**p* < .05; ***p* < .01; ****p* < .001

Table S5. Descriptive statistics and between-person correlations of depressive symptoms, quantity of social media activities, and social media experiences after excluding untrustworthy observations

| Variables | *n* | Correlation with depressive symptoms |
| --- | --- | --- |
| **Quantity social media activities** |  |  |
| Frequency of social media posting | 475 | -.033 |
| Time spent communicating online with friends | 475 | .077 |
| Time spent scrolling on social media | 475 | .090 |
| **Social media experiences** |  |  |
| Feedback preoccupation | 408 | .251*** |
| Perceived positivity of feedback | 408 | -.192*** |
| Friend support in online communication | 475 | -.230*** |
| Friend rejection in online communication | 475 | .420*** |
| Scrolling-induced fun | 475 | -.114* |
| Scrolling-induced insecurity | 475 | .409*** |

**p* < .05; ***p* < .01; ****p* < .001

Table S6. Partial correlations of depressive symptoms with quantity of social media activities and social media experiences, controlling for gender (boy/girl)

| Variables | *n* | Correlation with depressive symptoms |
| --- | --- | --- |
| **Quantity social media activities** |  |  |
| Frequency of social media posting | 479 | .012 |
| Time spent communicating online with friends | 479 | .105* |
| Time spent scrolling on social media | 479 | .112* |
| **Social media experiences** |  |  |
| Feedback preoccupation | 410 | .222*** |
| Perceived positivity of feedback | 410 | -.163*** |
| Friend support in online communication | 479 | -.204*** |
| Friend rejection in online communication | 479 | .411*** |
| Scrolling-induced fun | 479 | -.121** |
| Scrolling-induced insecurity | 479 | .450*** |

**p* < .05; ***p* < .01; ****p* < .001

Table S7. Partial correlations of depressive symptoms with quantity of social media activities and social media experiences, controlling for age


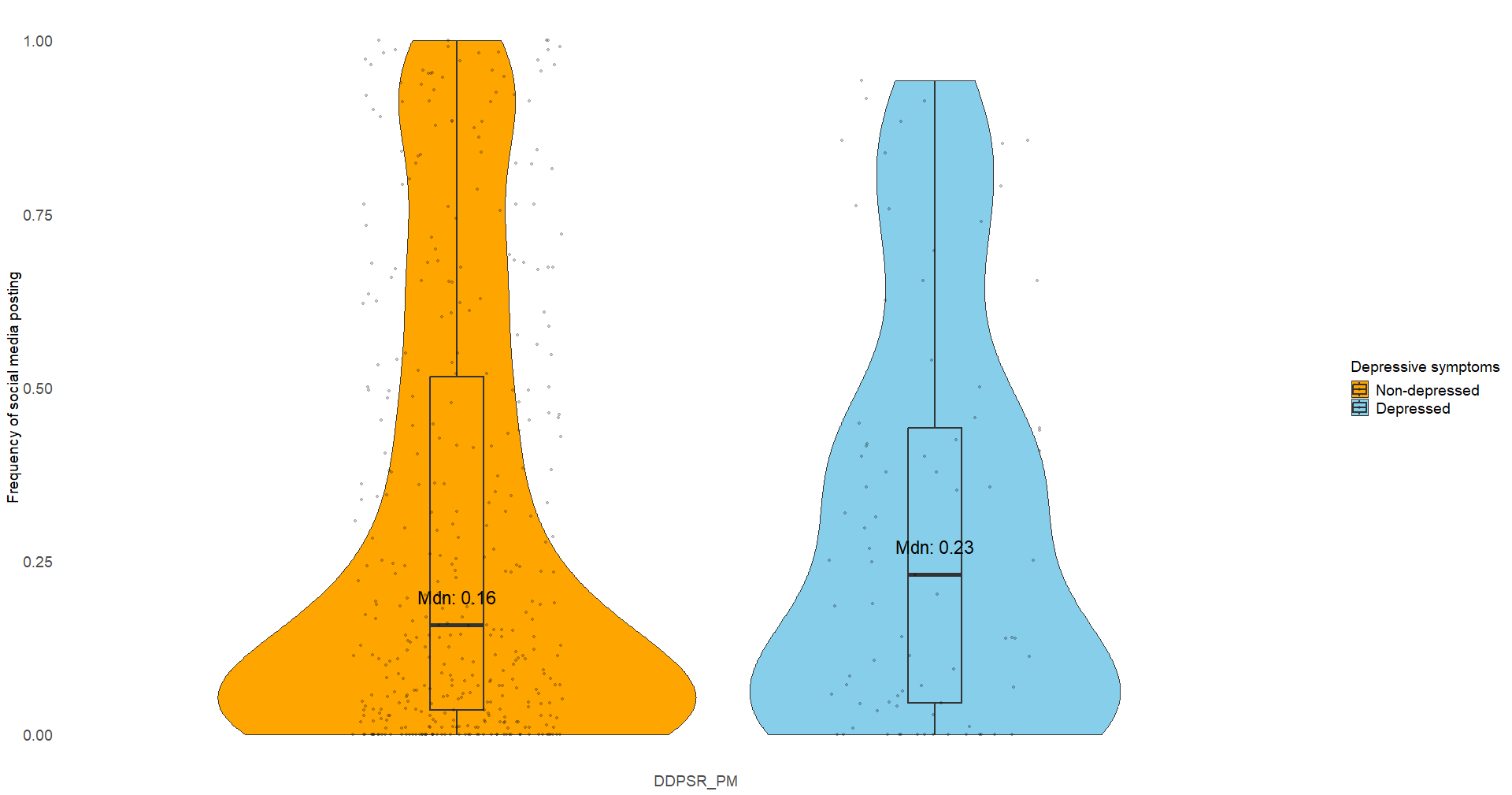


**Figure S1**

Violin plots visualizing the distribution of the frequency of social media posting among non-depressed (orange) and depressed (blue) adolescents including boxplots and median values. The data points reflect the person-mean scores across 100 days. Variations within the two subgroups are larger than the differences between the two groups.


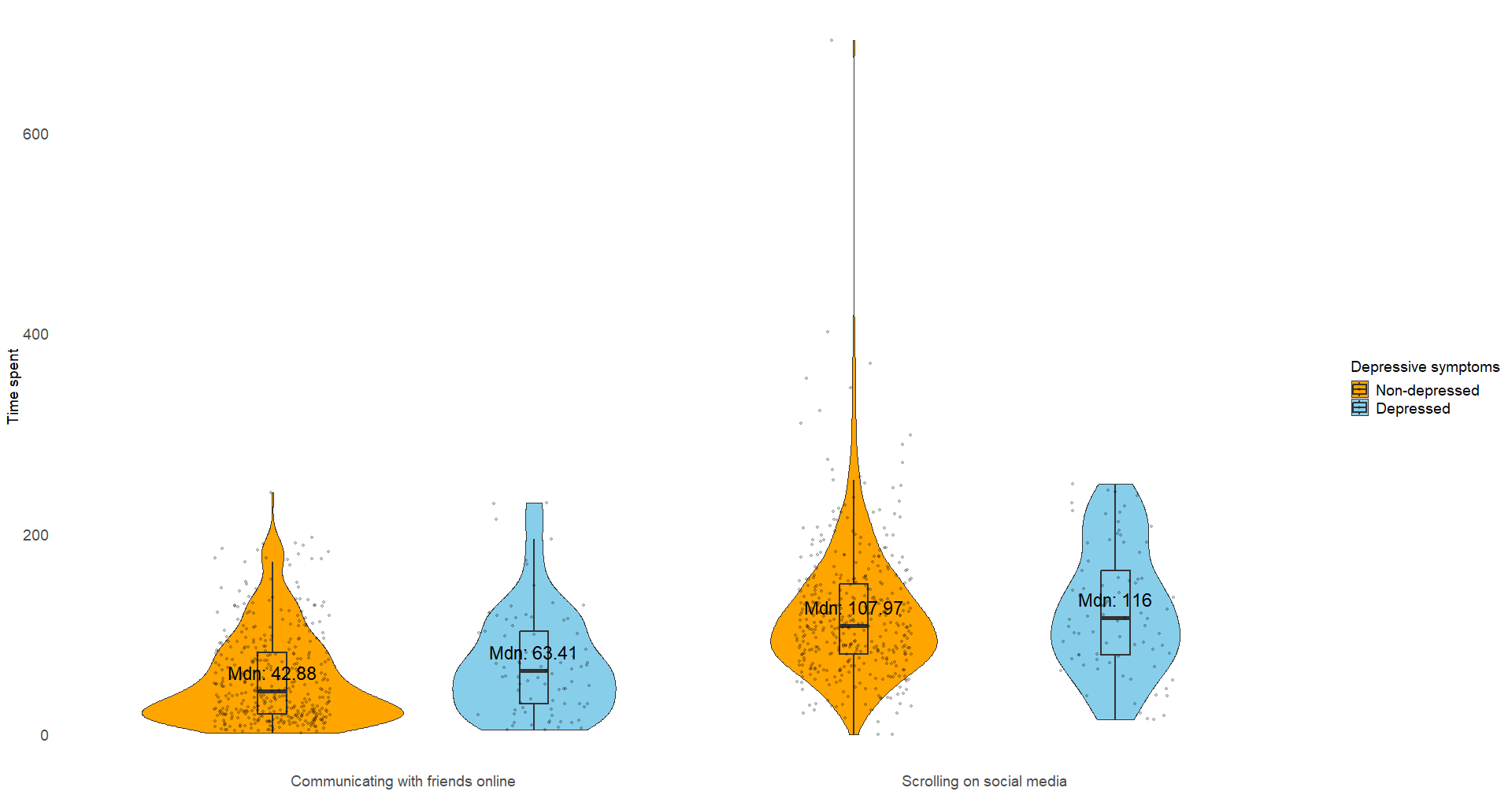


**Figure S2**

Violin plots visualizing the distribution of communicating with friends online (left) and the time spent scrolling on social media (right) among non-depressed (orange) and depressed (blue) adolescents including boxplots and median values. The data points reflect the person-mean scores across 100 days. For time spent communicating online with friends, variation within the two subgroups is larger than the differences between the two groups. For time spent scrolling on social media, differences between the two subgroups are larger than the variation within the two groups.

**
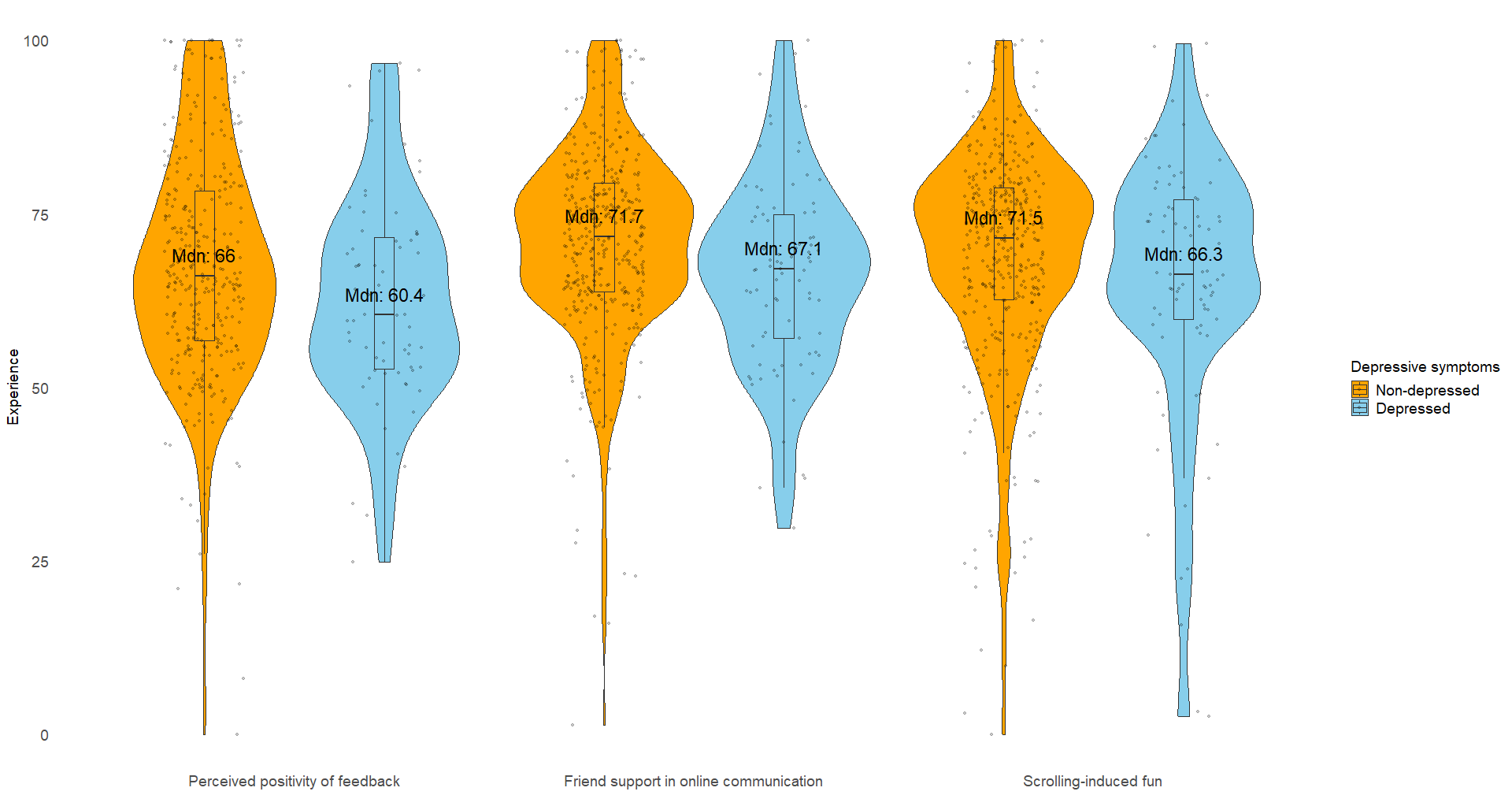
**

**Figure S3**

Violin plots visualizing the distribution of perceived positive of feedback, friend support in online communication, and scrolling-induced fun among the non-depressed (orange) and depressed adolescents (blue) including boxplots and median value. The data points reflect the person-mean scores across 100 days. Differences between the two subgroups are larger than the variation within the two groups.

**
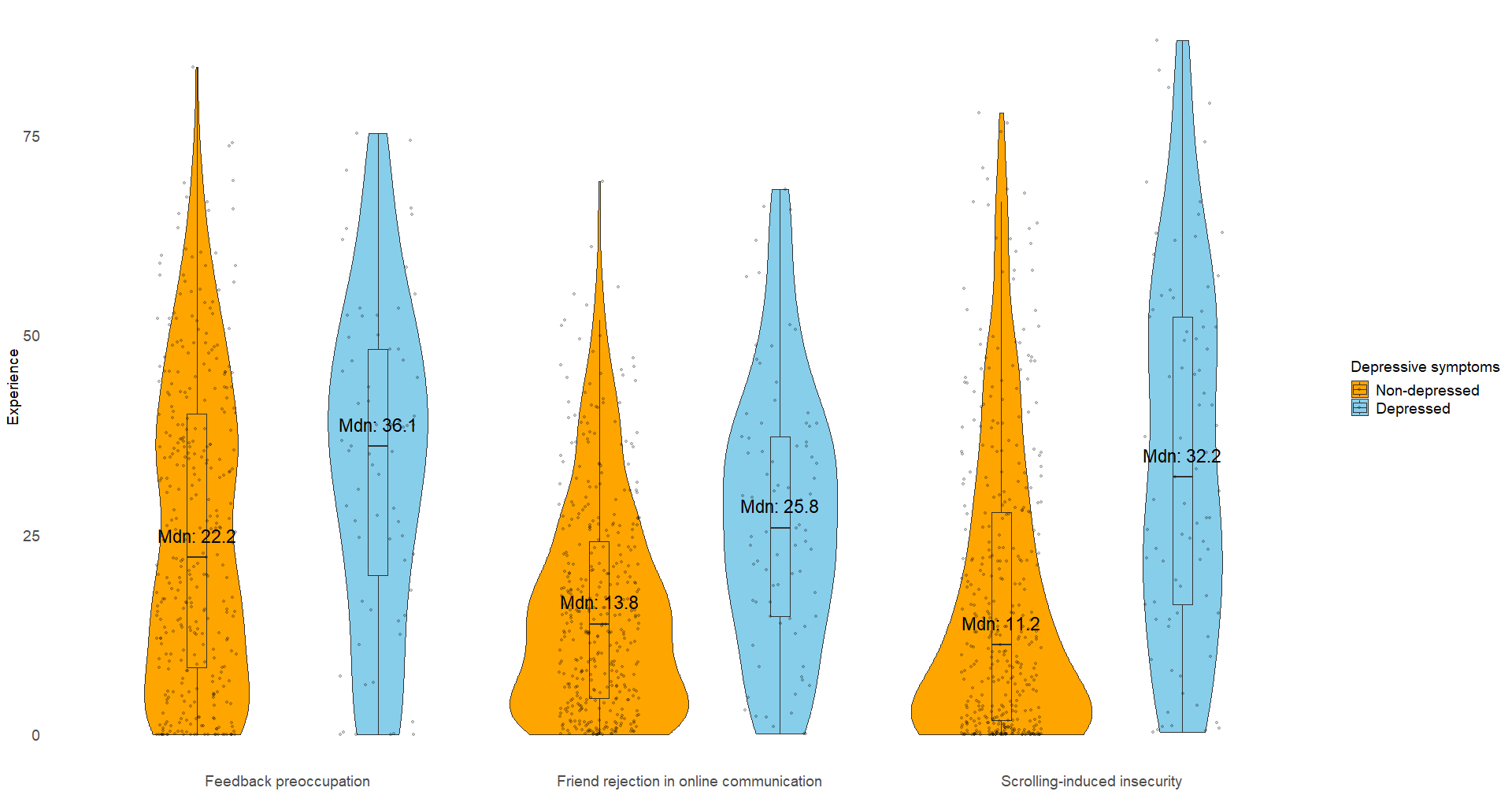
**

**Figure S4**

Violin plots visualizing the distribution of feedback preoccupation, friend rejection in online communication, and scrolling-induced insecurity among non-depressed (orange) and depressed (blue) adolescents including boxplots and median value. The data points reflect the person-mean scores across 100 days. Differences between the two subgroups are larger than the variation within the two groups.
